# Removal of Histamine from Fish Sauce by *Staphylococcus debuckii* sp. Isolated from Fermented Fish

**DOI:** 10.17113/ftb.61.03.23.7984

**Published:** 2023-09

**Authors:** Natthakan Rungraeng, Kazuhisa Ohtaguchi, Teerin Chysirichote

**Affiliations:** 1 Unit of Food Packaging and Biomaterials, School of Agro-Industry, Mae Fah Luang University, Muang, 57100 Chiang Rai, Thailand; 2 Department of Chemical Engineering, Tokyo Institute of Technology, 2-12-1 Ookayama, Meguro-ku, 152-8550 Tokyo, Japan; 3 Department of Food Engineering, School of Engineering, King Mongkut’s Institute of Technology Ladkrabang, Ladkrabang, 10520 Bangkok, Thailand

**Keywords:** fish sauce, salted fish, *Staphylococcus debuckii*, biogenic amine

## Abstract

**Research background:**

One of the issues in the production of fish sauce is the legal constraints on the concentration of histamine produced by bacteria during fermentation because it causes allergic reactions in humans. The goal of this study is therefore to eliminate histamine from the final product after fermentation to enhance the quality of fish sauce for consumer safety, manufacturer exportability and domestic sales.

**Experimental approach:**

The bacteria that grow in the histamine medium were isolated from the salted fish. Their ability to degrade histamine in the media with high NaCl content was tested. The bacterium with the highest histamine-degrading ability was identified and the histamine-degrading conditions were optimized, including the incubation temperature and the amount of NaCl in the medium. The regression equation was generated and tested using the local fish sauce in which different concentrations of histamine were added.

**Results and conclusions:**

Among the five bacteria isolated from the salted fish, the isolate with the greatest ability to degrade histamine was identified as *Staphylococcus debuckii* sp. The study of the capacity of the isolated bacteria to degrade histamine using the synthetic histamine broth (pH=7.0, *t*=25 °C and NaCl 25 % (*m*/*V*)) indicated that they were able to degrade up to 56 % of histamine. The optimization analysis showed that increasing the pH of the medium to 7.5 and lowering the incubation temperature to 20 °C could improve the histamine removal from 56 to 73 %. The generated regression model, validated by the experimental results of histamine removal from fish sauce, showed an acceptable error (not more than 10 %). *S. debuckii*, the isolated histamine-degrading bacteria, could be used as an inoculum to reduce histamine accumulated in fish products.

**Novelty and scientific contribution:**

The microbiological technique developed here can decrease the histamine concentration in the final product, fish sauce, to improve its quality in terms of food safety and satisfy the histamine regulation requirement. The findings of this study can also be used to treat other liquid foods that contain high concentrations of histamine.

## INTRODUCTION

Fish sauce is a common condiment in Thailand and southeastern countries in Asia (*1*), whose names and methods of production vary by region, such as Patis in the Philippines, Nouc-mam in Vietnam and Bakasang in Indonesia (*2*). It is generally prepared by mixing fish with salt in a 3:1 ratio and fermenting it at 30–35 °C for more than 6 months so that the hydrolysis of fish produces an odour (*3*).

Histamine (scombrotoxin) is a toxin that causes allergic reactions and is a worldwide concern due to the high consumption of seafood such as tuna, sardines, mackerel and abalone. The production of histamine toxins in such foods, which are sources of protein, is caused by the bacteria living on the fish and producing the enzyme decarboxylase, which breaks down the amino acid histidine (*4*) in seafood (*5*, *6*) by decarboxylation of histidine molecules. The amount of histamine found in seafood indicates the quality of those foods because it is related to the number of bacteria producing the enzyme, most of which can grow well at moderate temperatures between 20 and 40 °C (*7*), while a small amount of histamine was found in seafood when stored at 0–5 °C or under refrigeration storage (*8*-*10*). This indicated that the increase in histamine amount was caused by inappropriate preservation processes. Good-quality fish has less than 10 mg/kg histamine, while spoiled fish has 30 mg/kg. When a healthy person ingests at least 50 mg/kg histamine, the effects of histamine poisoning begin to manifest (*11*). Since histamine is resistant to heat and does not cause an unusual smell or taste of fish, it is not detectable by visual or sensory inspection.

Although heat destroys the bacteria that produce the toxin (*12*), histamine remains in the fish and can cause various allergic reactions in consumers (*13*). Reactions to scombrotoxin include rash, nausea and vomiting, diarrhoea or other symptoms. Many researchers are studying ways to reduce histamine production, such as using microorganisms (*14*, *15*), radiation at 2.5 and 5 kGy (*16*), oxygen scavengers (*17*, *18*) and modified atmosphere packaging (*19*) to inhibit the formation of bacterial biogenic amines. Even if histamine in fish sauce does not harm human health because it is taken in a small amount with each meal, its amounts in food are regulated by safety regulations in many countries, limiting the export of this product. The objectives of this research are the isolation and identification of the bacteria able to degrade histamine generated in salted fish. In addition, the histamine reduction ratio affected by the amount (*m*/*V*) of salt in fish sauce was studied to be applied to the downstream process of salted fish and fish sauce fermentation.

## MATERIALS AND METHODS

### Preparation of media

Four types of media were prepared in this research: histamine broth, histamine solid medium, trypticase soy broth (TSB) and trypticase soy agar (TSA). The histamine broth and solid medium were used to select from the local salted fish the bacteria that were able to grow in a histamine environment. TSB and TSA were prepared for growing and storing the isolated bacteria before the experiment of histamine removal. TSBs containing histamine and salt at various amounts were utilized to study the properties of the isolated bacteria.

Histamine broth was prepared by mixing (in % (*m*/*V*)): glucose (Kemaus, Cherrybrook, NSW, Australia) 0.1, yeast extract (Difco, Cockeysville, MD, USA) 0.2, NaCl (Kemaus) 0.5, histamine dihydrochloride (Thermo Fisher Scientific, St. Louis, MO, USA) 0.1, K_2_HPO_4_ (Kemaus) 0.05 and adjusting the pH to 7.0 using 0.1 M HCl (RCI Labscan, Bangkok, Thailand) and 0.1 M NaOH (Kemaus). The broth was autoclaved (HVA 85/110; Hirayama, Tokyo, Japan) at 121 °C at 0.10–0.12 MPa for 20 min. Histamine dihydrochloride was added through the 0.2-micron nylon syringe filter (Filtrex, Wayne, NJ, USA) to the medium after it was cooled down to room temperature.

Histamine solid medium was prepared by adding 2.0 % (*m*/*V*) agar (HiMedia, Mumbai, India) to the histamine liquid medium and mixing well before autoclaving under the same conditions as histamine broth. A 20-mL solution of histamine solid medium (45–50 °C) was poured into 86-mm diameter Petri dishes (T.S. Interlab Ltd., Bangkok, Thailand) and solidified at room temperature in the aseptic area. Histamine dihydrochloride was added through the 0.2-micron nylon syringe filter (Filtrex) to the medium.

TSB and TSA were prepared by suspending 40.0 g of the TSB and TSA powders (Difco) in 1.0 L of distilled water, respectively. The solutions were sterilized at 121 °C for 20 min using an autoclave (HVA 85/110; Hirayama, Tokyo, Japan). TSA was solidified in Petri dishes as described above.

### Selection of bacteria capable of digesting histamine

The fermented fish from different sources was bought from local markets. Each sample of 1.0 g different fermented fish was added to 10.0 mL histamine liquid medium and incubated at 30 °C for 7 days. Then, 0.1 mL fermented sample was streaked on the solid histamine medium and incubated at 30 °C for 7 days to obtain the individual colonies. Each collected colony growing on the solid medium was cultured in 50 mL TSB medium and shaken at 30 °C for 3 days. The cultured broth was then centrifuged at 8000×*g* (EBA 12; Hettich, Geldermalsen, The Netherlands) to obtain the isolated bacteria. Each bacterial cell was diluted using 50 mL sterilized water (the same volume as the removed TSB liquid) to achieve the same density of bacterial cells in the solution. Then, 250 μL bacterial suspension were added to 5 mL histamine broth to test its ability to destroy histamine under different salt amounts. They were cultured in TSB liquid medium containing 30 mg/L histamine dihydrochloride and 0, 10, 15, 20 and 25 % NaCl (*m*/*V*) and then incubated at 30 °C for 24 h. The maximum amount of NaCl was set at 25 % because of its solubility. Histamine content in the fermented liquid medium and the number of viable bacteria were determined. The most histamine-degrading bacteria were identified using the 16S rDNA gene sequencing method.

### Characterization of the isolated bacteria

The selected bacteria that had the highest histamine-degrading capacity were characterized. Histamine liquid media (*γ*(histamine)=30 mg/L) used in this experiment were prepared by adding NaCl at 5, 15 and 25 % (*m*/*V*) and adjusting the pH values using 0.1 M HCl and 0.1 M NaOH solutions to 5.5, 6.5 and 7.5. Once modified, all media were sterilized and subsequently their pH value was checked. The prepared media were inoculated with one colony of the isolated bacteria and incubated at 20–50 °C. The experimental design was conducted using a Box-Behnken design with triplicate replication. The regression analysis was conducted to develop the model for predicting the histamine removing capacity of the isolated bacteria according to the NaCl amount, incubation temperature and pH of the medium. The regression model was validated against the experimental histamine removal in the fish sauce and the predicted value was calculated using the following equation:

HR_predicted_=aX_1_+bX_2_+cX_3_+dX_1_X_2_+eX_1_X_3_+fX_2_X_3_+gX_1_X_2_X_3_ /1/

where HR is histamine removal, X_1_ is the pH of the medium, X_2_ is incubation temperature (°C), X_3_ is NaCl in % (*m*/*V*) and a, b, c, d, e, f and g are coefficients.

Five commercial fish sauces purchased from the supermarket were evaluated for pH value and NaCl content. One fish sauce contaminated with the highest histamine concentration was selected for validation of the obtained regression model. Besides the original concentration of histamine in the fish sauce, we added histamine dihydrochloride to it to obtain a total of five different histamine concentrations. A volume of 5.0 mL of each concentration was inoculated with 250 μL of 3-day precultured bacteria in TSB and incubated at 20 °C (the optimized temperature obtained from the previous experiment) for 24 h. The concentrations of histamine before and after incubation were analyzed to evaluate the percentage of histamine removal.

### Determination of viable bacteria

The sample in liquid medium was diluted 10, 100 and 1000 times, and then 1.0 mL of the diluted sample was cultured in TSA agar by the pour plate method. After the solid medium was set, it was incubated at 30 °C for 72 h, and the colonies were counted and expressed in CFU/g (*20*). Five replicates per dilution were conducted per test.

### Quantification of histamine

An enzyme immunoassay using Ridascreen Histamine (R-Biopharm, Darmstadt, Germany) No. R1605 (*21*) was used to determine histamine concentration. Briefly, the sample was boiled in water to extract the histamine from the sample. After centrifugation (EBA 12; Hettich), the supernatant was derivatized into N-acyl-histamine using an acylation reagent. Free acylated histamine and bound histamine bind with the antibody (I) added to the sample. After washing, another antibody (II) labelled with peroxidase was added to create the antibody-histamine complexes (a blue product). Finally, the stop solution was added to change the colour from blue to yellow. The absorbance was measured at 450 nm using a spectrophotometer (Thermo Spectronic, Genesys Ios UV-Vis; Thermo Fisher Scientific, Waltham, MA, USA). It was inversely proportional to the histamine concentration in the sample. The results of histamine concentration were used to calculate the percentage of removed histamine in the experimental with the following equation:

*γ*(histamine)_removed_={[*γ*(H_0_)-*γ*(H_t_)]/*γ*(H_0_)}·100 /2/

where *γ*(H_0_) is the initial and *γ(*H_t_) the remaining concentration of histamine in the sample (in mg/L) after incubation.

### Determination of sodium chloride content

NaCl amount was determined according to the AOAC method 937.09 (*22*). A volume of 5.0 mL of the sample was diluted with distilled water to 100 mL, and 1.0 mL diluted sample was mixed with 10.0 mL of 0.1 M AgNO_3_ (Fisher Chemical, Loughborough, UK) and 10.0 mL concentrated HNO_3_ (Loba Chemie, Mumbai, India). The mixture was then gently boiled for 30 min. After cooling to room temperature, the mixture was added to 50.0 mL of distilled water and 5.0 mL of ammonium iron(III) sulfate (Hach, Loveland, CO, USA). It was titrated with 0.1 M KSCN (Loba Chemie Loba Chemie). The salt amount was calculated and presented as % (*m*/*V*).

### Statistical analysis

The experiment was conducted with three replicates and the results were reported as the average value±standard deviation (S.D.). The results were analyzed using analysis of variance (ANOVA) and the Tukey’s test for comparison. The significant difference was analyzed at p≤0.05. All statistical analyses were performed using the Minitab 19 program (*23*). Regression analyses of combinations of significant single and interaction variables were considered to establish a stronger correlation between the input and output parameters.

## RESULTS AND DISCUSSION

### Isolation of histamine-degrading bacteria

**Fig. 1:**
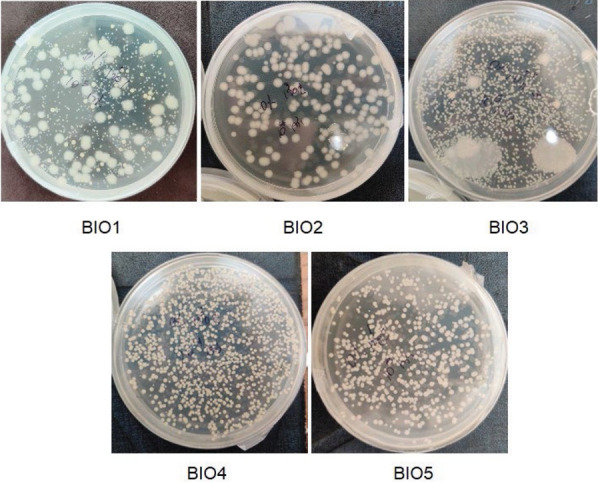
Bacteria isolated from the salted fish. BIO1-BIO5=bacteria codes as shown in Table 1

**Fig. 2:**
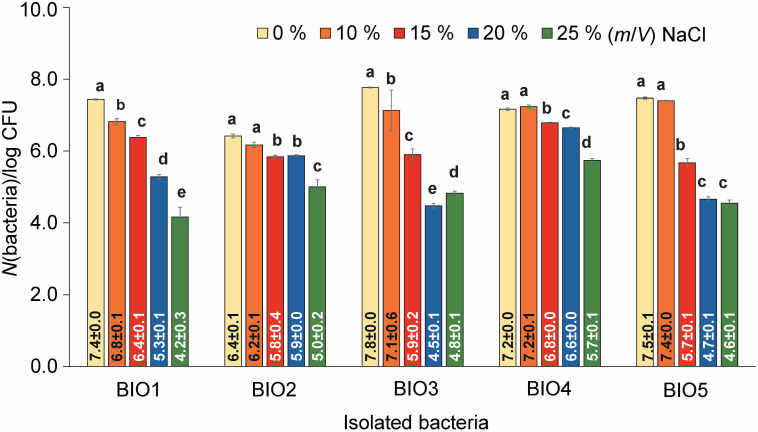
Viable count of the bacteria isolated from the salted fish cultivated on *γ*(histamine)=30 mg/L broth containing different amounts of NaCl. Different letters for the same bacteria show significant difference (p≤0.05). BIO1-BIO5=bacteria codes as shown in Table 1

**Fig. 3:**
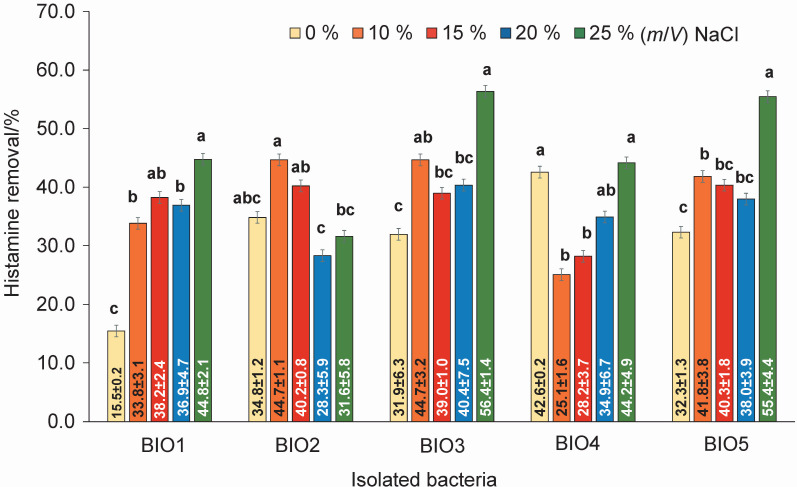
Histamine removal by the bacteria isolated from the salted fish cultivated on *γ*(histamine)=30 mg/L broth containing different amounts of NaCl. Different letters for the same bacteria showed significant difference (p≤0.05). BIO1-BIO5=bacteria codes as shown in Table 1

Five bacteria were isolated from ten salted fish products, purchased from different local markets, using the histamine solid medium (*w*(histamine)=30 mg/kg). They were coded as BIO1–5, as shown in Fig. 1. Even if they were able to grow on the media containing NaCl up to 25 %, the bacterial count was higher in the media containing a lower NaCl amount, as shown in Fig. 2. The highest histamine-removing capacity among the isolated bacteria (Fig. 3) obtained from the histamine medium containing 25 % NaCl was found in bacteria BIO3 and BIO5 at 56.4 and 55.4 %, followed by bacteria BIO2 and BIO4 at 31.6 and 44.2 % (*m*/*V*). However, bacteria BIO2 mostly degraded histamine in the medium containing 10 % NaCl. Since BIO1, BIO3 and BIO5 showed high capacities of histamine removal in the media containing NaCl in the range of 10–25 %, which are usually used in the production of fish sauce, they were identified, as shown in Table 1, as *Staphylococcus debuckii*, found in animal environments such as bovine milk (*24*), and *Staphylococcus condimenti*, found in soy sauce mash (*25*). *S. condimenti* was found as an infection bacterium associated with catheter-related bacteremia (*26*). The isolated bacterium *S. debuckii* was selected to evaluate the optimum conditions for histamine degradation.

**Table 1:** List of histamine-degrading bacteria isolated from salted fish products

Code	Organism identified	Strain	Strain identified/%	Accession
BIO 1	*Staphylococcus condimenti*	DSM11674	99	CP015114
BIO 2	N/A	N/A	N/A	N/A
BIO 3	*Staphylococcus debuckii*	SDB2975	99	MK121623
BIO 4	N/A	N/A	N/A	N/A
BIO 5	*Staphylococcus debuckii*	SDB2975	100	MK121623

*N/A=not analyzed

### Characterization of the isolated bacterium S. debuckii

**Fig. 4:**
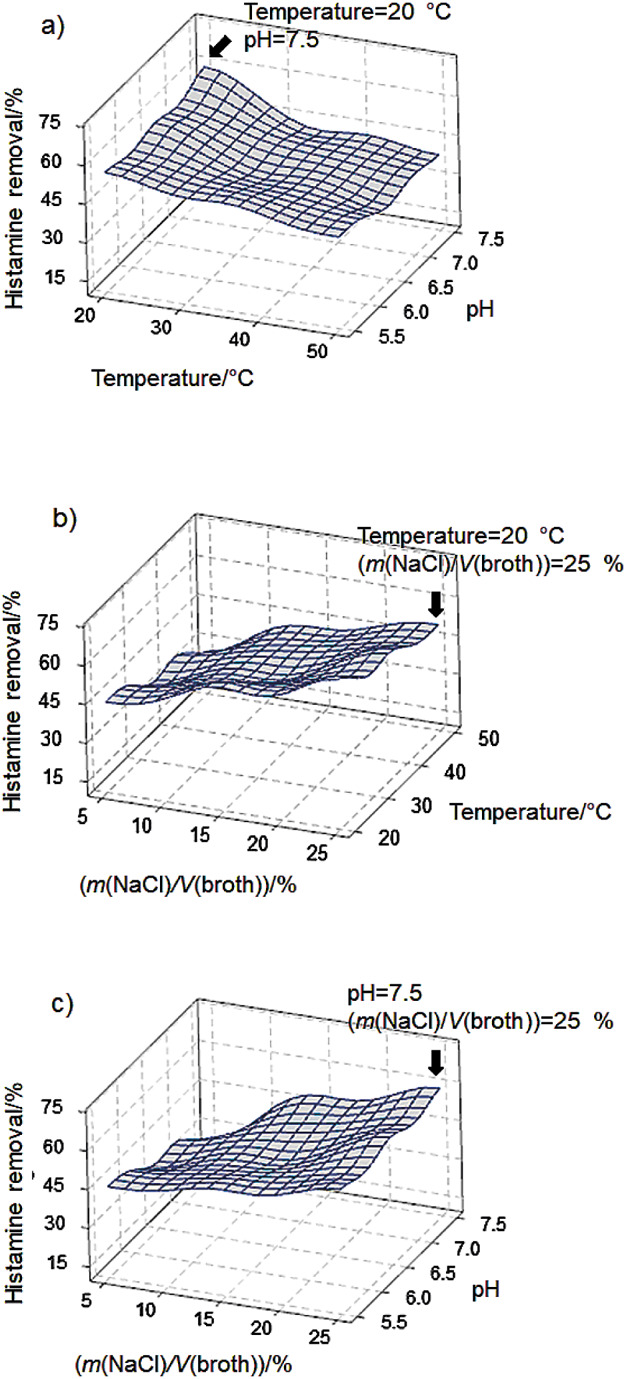
Surface plots of histamine removal of *S. debuckii* isolated from salted fish: a) effect of incubation temperature and pH of the medium, b) effect of NaCl amount and incubation temperature, c) effect of NaCl amount and pH of the medium

**Fig. 5:**
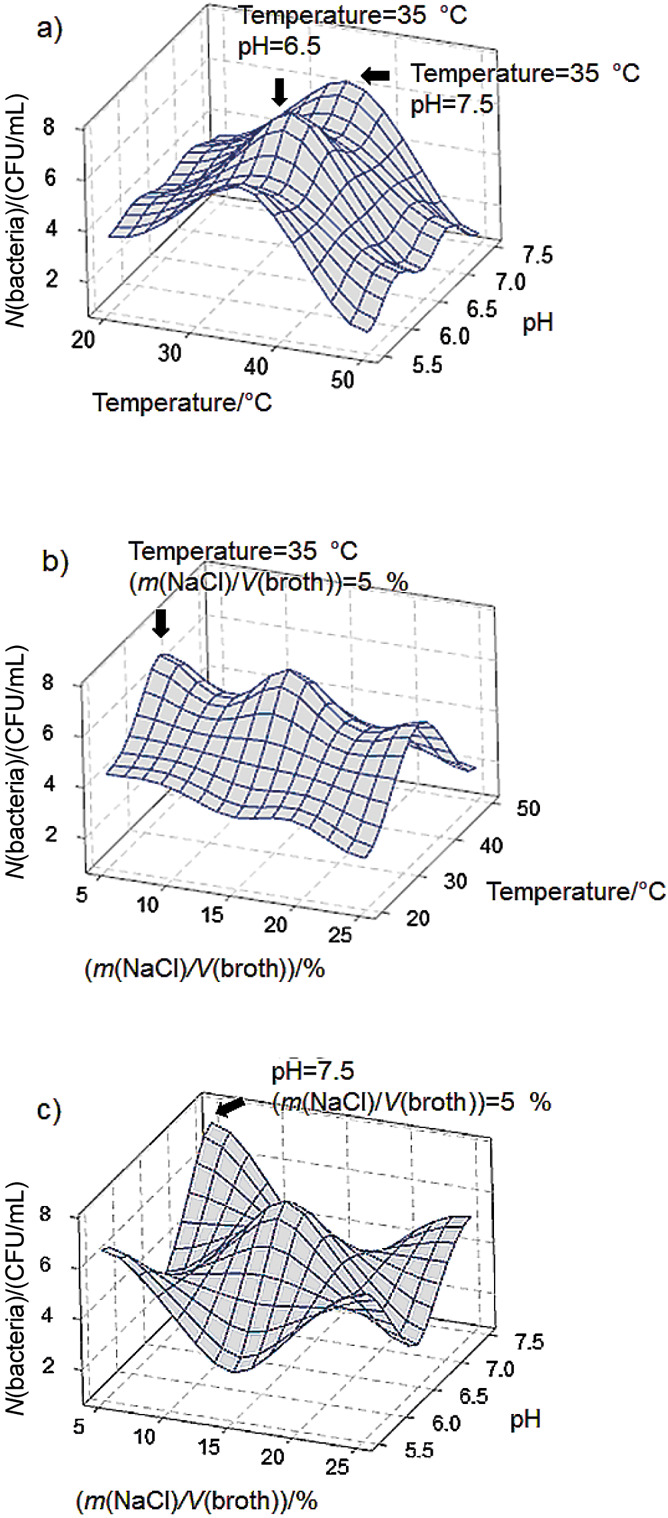
Surface plots of viable bacterial count of *S. debuckii* isolated from salted fish: a) effect of incubation temperature and pH of the medium, b) effect of NaCl amount and incubation temperature, c) effect of NaCl amount and pH of the medium

The surface plots of histamine removal as a response to the change of pH of the medium and incubation temperature (Fig. 4a) showed that low temperature (20 °C) and high pH (7.5) were more suitable for *S. debuckii* to degrade histamine. Also, incubating *S. debuckii* cultivated in a high percentage of NaCl (25 % (*m*/*V*)) enhanced the histamine removal, as shown in Fig. 4b and Fig. 4c. In contrast to the number of viable bacteria, it was found that *S. debuckii* grew well at 35 °C, as shown in Fig. 5a and Fig. 5b and at low NaCl amount (5 % (*m*/*V*)), shown in Fig. 5b and Fig.5c. The high pH of the medium (pH=7.5) enhanced both the histamine elimination and the number of viable bacteria, while the percentage of NaCl in the solution was positive for the former but negative for the latter as compared between Fig. 4c and Fig. 5c.

**Fig. S1:**
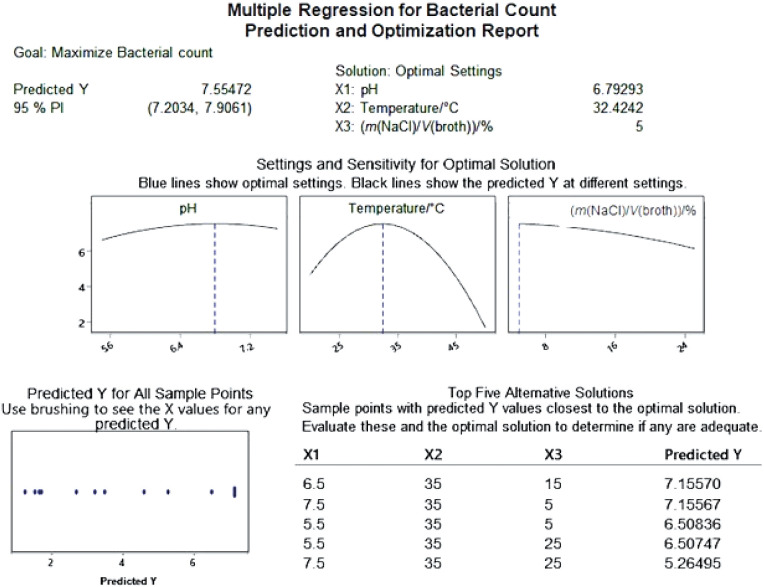
Optimization response of multiple regression for maximum bacterial count

**Fig. S2:**
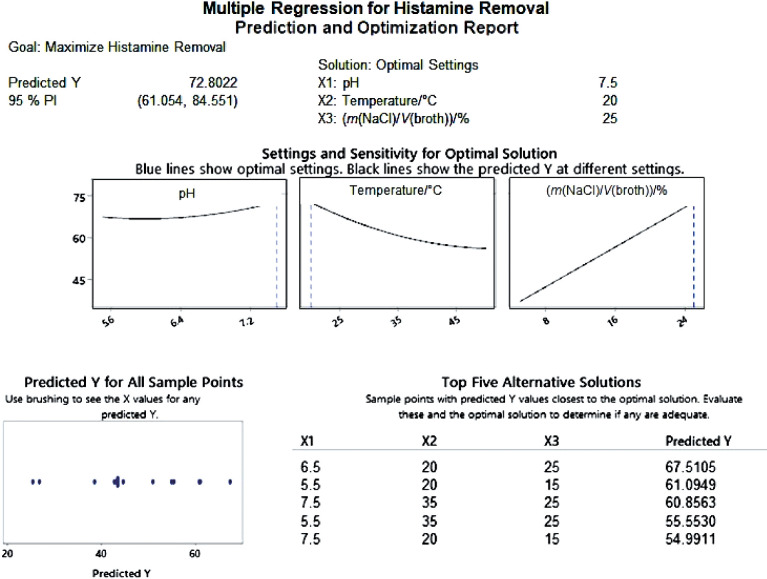
Optimization response of multiple regression for maximum histamine removal

The optimization analysis showed that *S. debuckii* grew well (7.6 log CFU/g) during cultivation in the medium containing 5 % (*m*/*V*) NaCl, pH=6.8, at 32.4 °C as shown in Fig. S1, while its capacity for histamine removal was higher (72.8 %) in the medium containing a higher mass per volume ratio of NaCl (25 %) with pH=7.5 and incubation at lower temperature (20 °C), as shown in Fig. S2. Compared to other research using *Bacillus* and *Lactobacillus*, shown in Table 2 (*27*-*31*), in this work histamine was reduced in the medium containing a high percentage of NaCl, because the *Staphylococcus* strain is generally tolerant to high salt stress (*32*).

**Table 2:** Histamine degradation using different methods

Added compound	Histamine degradation/%	(*m*(NaCl)/*V*(solution))/%	Reference
Fermented rice bran in fish sauce	50-58	-	(*27*)
*Allium sativum* essential oil during fermenting fish sauce	49	-	(*28*)
*Bunium persicum* essential oil during fermenting fish sauce	42	-	(*28*)
Wheat bran during fermenting fish sauce	13.3	-	(*29*)
*Bacillus polymyxa* D05-1 in histamine solution	100 0	5 >5	(*30*)
*Lactobacillus plantarum* in de Man, Rogosa and Sharpe broth	100 70 50	0-6 (broth) 9 (broth) 12 (broth)	(*31*)
*S. debuckii* in trypticase soy broth with histamine	56 75	25 25 (pH=7.5, *t*=20 °C)	This study

### Regression model for predicting the histamine-removing capacity of the isolated bacterium S. debuckii from fish sauce

The results of viable cell number and histamine-reducing capacity of *S. debuckii* cultivated in the media with different conditions of pH, temperature and NaCl mass per volume ratio were used to analyze the important factors affecting the number of viable bacteria and the percentage of histamine removal, as shown in Table S1 and Table S2, respectively. The viable count was significantly affected by the pH value of the medium, while the pH value and incubation temperature were the main factors affecting histamine removal. Moreover, the interaction effects of pH value×temperature, temperature×NaCl and pH×temperature×NaCl influenced the histamine removal.

According to the regression analysis, the following response equation was generated using only the statistically significant terms in Table S3, with the acceptable R^2^, adjusted R^2^ and R^2^ for prediction as 98.9, 98.7 and 98.5 %, respectively.

HR_experimental_=7.28X_1_+2.89X_2_+1.23X_3_-0.528X_1_X_2_-0.106X_2_X_3_+0.0163X_1_X_2_X_3_ /3/

where X_1_ is the pH of the medium, X_2_ is incubation temperature (°C) and X_3_ is NaCl in % (*m*/*V*).

The p-value of the lack-of-fit obtained from the regression model (p≤0.05) shows that the obtained model accurately fits the data. It also shows that in the pH range of 5.5–7.5, incubation temperature of 20–50 °C and NaCl mass per volume ratio from 0 to 25 %, the positive coefficients of pH (X_1_), incubation temperature (X_2_) and NaCl in % (*m*/*V*) (X_3_) explained how their increases can increase histamine removal. The interactions pH×temperature and temperature×NaCl had a negative effect on histamine removal and were indicated as the negative coefficients in the equation. In contrast, the individual factors, including pH of the medium, incubation temperature and NaCl amount, were positive for the histamine removal by *S. debuckii.*

### Validation of the prediction model for histamine degradation degree

**Fig. 6:**
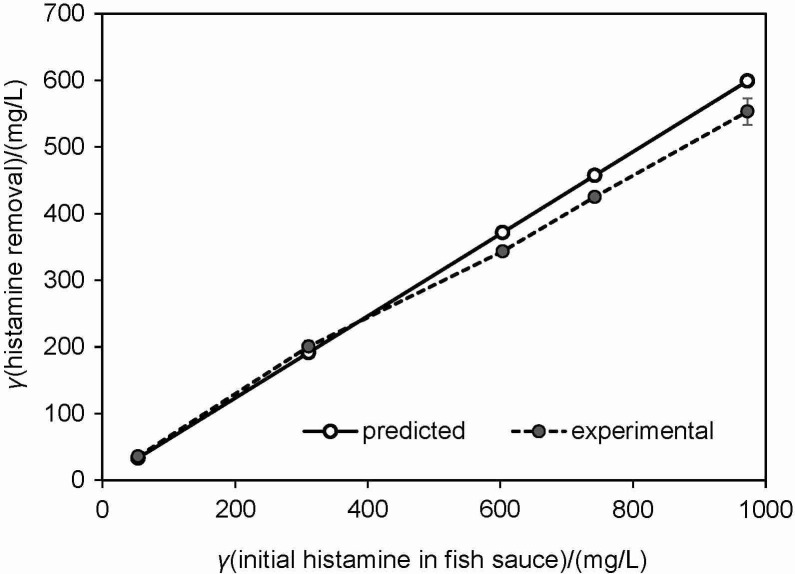
Amount of removed histamine in fish sauce containing different initial histamine concentrations obtained from the experiment and the prediction model

A regression model (Eq. 2) established to estimate the percentage of histamine removal depending on the pH of the medium (5.5–7.5), incubation temperature (20–50 °C) and NaCl amount (5–25 % (*m*/*V*)) was validated using fish sauce containing various histamine concentrations. The pH and percentage of NaCl in the fish sauce used to validate the model were 5.53 and 24 %, respectively. Discrepancies between the model-calculated values and the experimental ones of histamine removal percentage were around 4.5–8.3 % because of the different compositions of fish sauce samples, such as various kinds and amounts of amino acids, fatty acids and minerals (*2*, *33*, *34*), which might be an energy source for bacterial metabolism. A comparison of the concentrations of reduced histamine shown in Fig. 6 indicated that the experimental result of histamine removal from the fish sauce containing more than 360 mg/L histamine was lower than the model-predicted one.

## CONCLUSIONS

*Staphylococcus debuckii* was isolated from salted fish in this study. It was found to degrade histamine by up to 56 % in the histamine broth containing 25 % NaCl (*m*/*V*) at pH=7.0 and incubated at 25 °C. To enhance the histamine-degrading capacity of the bacteria, the optimum conditions were found to be 25 % NaCl (*m*/*V*), pH=7.5 and 20 °C. The bacteria removed 73 % of histamine under the optimum conditions. The statistical analysis showed the single and interaction effects of pH of the medium, incubation temperature, pH×temperature, temperature×NaCl amount and pH×temperature×NaCl amount. The validation of the prediction model using local fish sauce showed an agreed-upon acceptance of 8.3 % error. The isolated *S. debuckii* bacteria was predominant in the degradation of histamine in the high-salt products. This discovery is useful for the manufacturing of fish sauce since it helps to decrease the accumulated histamine that is often produced during fish fermentation.

## Appendix

**Table S1:** Analysis of variance: bacterial count *versus* pH of the medium, NaCl amount and incubation temperature

Source	DF	Adj SS	Adj MS	F**-**value	p**-**value
Regression	7	930.22	132.888	24.95	0.000
pH	1	85.54	85.538	16.06	0.000*
Temperature/°C	1	1.11	1.108	0.21	0.651
(*m*(NaCl)/*V*(broth))/%	1	2.67	2.666	0.50	0.484
pH×temperature/°C	1	5.42	5.421	1.02	0.319
pH×(*m*(NaCl)/*V*(broth))/%	1	4.03	4.032	0.76	0.390
Temperature/°C×(*m*(NaCl)/*V*(broth))/%	1	0.77	0.768	0.14	0.706
pH×temperature/°C×(*m*(NaCl)/*V*(broth))/%	1	1.25	1.254	0.24	0.630
Error	38	202.37	5.326		
Total	45	1132.59			

*Significant factor of *N*(viable cells)/log CFU at 95 % confidence level (*N*=3)

**Table S2:** Analysis of variance: predicted histamine removal *versus* pH of the medium, NaCl amount and incubation temperature

Source	DF	Adj SS	Adj MS	F-value	p-value
Regression	7	104447	14921.1	494.57	0.000
pH	1	3548	3548.2	117.61	0.000*
Temperature/°C	1	1554	1553.7	51.50	0.000*
(*m*(NaCl)/*V*(broth))/%	1	110	110.2	3.65	0.064
pH×temperature/°C	1	2032	2031.6	67.34	0.000*
pH×(*m*(NaCl)/*V*(broth))/%	1	58	57.8	1.92	0.174
Temperature/°C×(*m*(NaCl)/*V*(broth))/%	1	258	258.0	8.55	0.006*
pH×temperature/°C×(*m*(NaCl)/*V*(broth))/%	1	262	261.7	8.67	0.005*
Error	38	1146	30.2		
Total	45	105594			

*Significant factor of the percentage of histamine removal at 95 % confidence level (*N*=3)

**Table S3:** Analysis of variance: concentration of removed histamine *versus* pH of the medium, NaCl amount and incubation temperature

Source	DF	Adj SS	Adj MS	F-value	p-value
Regression	6	104390	17398.3	563.46	0.000
pH	1	3508	3507.6	113.60	0.000*
Temperature/°C	1	1503	1502.7	48.67	0.000*
(*m*(NaCl)/*V*(broth))/%	1	284	284.3	9.21	0.004*
pH×temperature/°C	1	1974	1974.2	63.93	0.000*
Temperature/°C×(*m*(NaCl)/*V*(broth))/%	1	399	398.8	12.91	0.001*
pH×temperature/°C×(*m*(NaCl)/*V*(broth))/%	1	456	456.2	14.77	0.000*
Error	39	1204	30.9		
Lack-of-fit	7	385	55.0	2.15	0.067
Pure error	32	819	25.6		
Total	45	105594			

*Significant factor of the percentage of histamine removal at 95 % confidence level (*N*=3)
